# Causal Effects of Circulating Micronutrients on Cognitive Function: Evidence From a Mendelian Randomization Study

**DOI:** 10.1002/brb3.70488

**Published:** 2025-04-22

**Authors:** Sen‐fu Yuan, Chang‐hai Long, Xu Zhang, Mi Yuan, Jun Li, He‐gang Wu

**Affiliations:** ^1^ Department of Pathology The First People's Hospital of Yibin Yibin China

**Keywords:** cognitive function, Mendelian randomization, micronutrients

## Abstract

**Background:**

Cognitive impairment is a growing concern worldwide, driven by an aging population. Emerging evidence suggests that micronutrients may play critical roles in maintaining cognitive health and preventing neurodegeneration. However, the causal relationships between specific micronutrients and cognitive function remain unclear.

**Methods:**

This study employed a two‐sample Mendelian randomization (MR) approach to investigate the causal effects of 16 circulating micronutrients on cognitive function. Genetic variants associated with micronutrient levels were used as instrumental variables (IVs), and cognitive outcomes, including reaction time, cognitive performance, prospective memory, and fluid intelligence, were assessed using publicly available genome‐wide association study (GWAS) datasets. Sensitivity analyses were conducted to evaluate heterogeneity, pleiotropy, and robustness of the findings.

**Results:**

MR analysis revealed potential positive effects of β‐carotene and phosphorus on reaction time, reflecting faster cognitive responses. Vitamin E was positively associated with cognitive performance, while vitamin B6 had a negative effect. Selenium was positively correlated with fluid intelligence, whereas elevated vitamin A1 levels were associated with reduced fluid intelligence. No significant associations were observed for other micronutrients across the cognitive domains assessed.

**Conclusion:**

This study highlights the roles of specific micronutrients, like β‐carotene, phosphorus, selenium, and vitamin E, in cognitive health, while excessive vitamin A1 and B6 may be harmful, warranting further investigation.

## Graphical abstract

Overview of the study design. Genetic variations used as instrumental variables (IVs) must satisfy three criteria: 1) The variation must show a strong association with the exposures. 2) It should remain unaffected by confounders linked to the outcome. 3) Its influence on the outcome should occur exclusively through the exposure pathway, without involvement of other biological mechanisms.

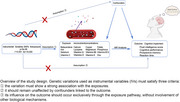

## 1 Background

With global population aging, the number of individuals with cognitive impairment continues to rise (Petersen et al. 2018). Cognitive impairment primarily manifests as declines in memory, judgment, comprehension, and reasoning abilities. Mild cognitive impairment (MCI) not only significantly affects patients’ quality of life and daily functioning (Aranda et al. 2021) but is also considered a potential risk factor for dementia progression. It is estimated that over 35% of individuals with MCI progress to dementia annually (Mitchell and Shiri‐Feshki 2009). Currently, approximately 6.9 million Americans aged 65 and older live with dementia, a number expected to reach 13.8 million by 2060 (2024). This trend imposes severe psychological and financial burdens on patients and their families while presenting significant challenges to healthcare systems and economies (Livingston et al. 2017). Given the lack of effective treatments for cognitive impairment and dementia, identifying and addressing modifiable risk factors has become a pressing priority.

The etiology of MCI is complex and multifactorial. Recent studies indicate that micronutrients play a critical role in maintaining cognitive function and preventing neurodegenerative diseases (Morris et al. 2018). For instance, research by Engelhart et al. (2002) found that higher intake of vitamin C, vitamin E, and β‐carotene was associated with a reduced risk of cognitive impairment. Similarly, Devarshi et al. (2023) reported that higher consumption of vitamin C, D, E, folate, and carotenoids was associated with better cognitive abilities. Furthermore, Lin et al. (2022) observed that higher baseline calcium levels might be linked to cognitive decline, and Garrido‐Dzib et al. (2023) noted that MCI and dementia could be associated with lower folate and calcium intake.

However, not all studies have reached consistent conclusions. Some failed to find significant associations between micronutrients and cognitive function or even reported conflicting results (Crichton et al. 2013). For instance, Peacock et al. (2000) and Sato et al. (2006) found minimal correlation between vitamin C intake and cognitive function, and Jama et al. (1996) observed no relationship between vitamin C or E intake and cognitive performance. In addition, studies on β‐carotene yielded no significant findings (Peacock et al. 2000; McNeill et al. 2011). These results suggest that the relationship between micronutrients and cognitive decline may be complex and multifaceted.

Most studies examining micronutrients have been observational, making it difficult to fully eliminate confounding factors and reverse causality. With advancements in genomics and genetic epidemiology, many genetic variants associated with human diseases have been identified. Mendelian randomization (MR) is an epidemiological technique that leverages genetic data to assess causal relationships. Since the allocation of alleles at conception is random and unaffected by environmental or unknown confounders, MR uses genetic variants as instrumental variables (IVs) to effectively address confounding and reverse causality issues, thereby providing more reliable causal inference (Lawlor et al. 2008; Sanderson et al. 2019).

This study utilized a two‐sample MR approach, employing genetic variants associated with micronutrient levels as IVs, to investigate the potential causal relationships between 16 micronutrients and cognitive impairment. By elucidating these relationships, we aim to provide scientific evidence for developing effective nutritional interventions to prevent or mitigate cognitive decline.

## 2 Methods

### 2.1 Study Design

Our study followed the MR‐STROBE guidelines for reporting MR studies (Skrivankova et al. 2021). A two‐sample MR design based on single nucleotide polymorphisms (SNPs) was employed to infer causal relationships between circulating micronutrients and cognitive impairment. SNPs, randomly allocated at conception and independent of environmental factors, minimize confounding effects. The validity of the genetic instruments relied on three core principles:

- Assumption 1: The selected SNPs were strongly associated with the exposure (micronutrient levels).
- Assumption 2: The selected SNPs were not associated with confounding factors affecting the outcome.
- Assumption 3: The selected SNPs influenced the outcome only through the exposure, not through other pathways.

This study utilized publicly available data, negating the need for ethical approval or informed consent. Table S1 provides detailed information on exposure and outcome data.

### 2.2 Genetic Instrument Selection

We searched PubMed and the IEU OpenGWAS catalog (https://gwas.mrcieu.ac.uk [accessed on November 10, 2024]) for published genome‐wide association studies (GWAS) on circulating micronutrient levels and obtained summary statistics for 16 micronutrients: β‐carotene (Ferrucci et al. 2009), calcium (O'Seaghdha et al. 2013), copper, selenium, zinc (Evans et al. 2013), folate, vitamins B12 (Grarup et al. 2013), iron (Bell et al. 2021), lycopene (D'Adamo et al. 2016), magnesium (Meyer et al. 2010), phosphorus (Kestenbaum et al. 2010), vitamins A1 (Mondul et al. 2011), B6 (Hazra et al. 2009), C (Zheng et al. 2021), D (Jiang et al. 2018), and E (Major et al. 2011). Due to the lack of available GWAS data, other micronutrients like chromium and molybdenum were not included in the analysis.

To ensure strong relevance, SNPs with *p* < 5E^−08^ were selected. Weak instruments were avoided by requiring an *F*‐statistic > 10. Table S2 lists the SNPs associated with micronutrient levels.

### 2.3 Cognitive Function Data Sources

This study examined the relationships between circulating micronutrients and four cognitive outcomes: fluid intelligence, cognitive performance, prospective memory, and reaction time.

Summary statistics for cognitive performance were obtained from GWAS conducted by the Social Science Genetic Association Consortium (SSGAC) (Lee et al. 2018). These studies combined data from the Cognitive Genomics Consortium and the UK Biobank and were accessed via the IEU Open GWAS Project (GWAS ID: ebi‐a‐GCST006572) at https://gwas.mrcieu.ac.uk. In addition, data on fluid intelligence, prospective memory, and reaction time were retrieved from the IEU Open GWAS Project (https://gwas.mrcieu.ac.uk) (GWAS IDs: ukb‐b‐5238, ukb‐b‐4282, ukb‐b‐16287) on November 12, 2024 (Haworth et al. 2019). Tables S3, S4, S5, and S6 list the SNPs associated with cognitive function.

### 2.4 Sensitivity Analysis

Cochrane's *Q* test was used to assess heterogeneity. When evaluating heterogeneity using Cochrane's *Q* test, a *p* value less than 0.05 indicates significant heterogeneity. In such cases, we prioritize the use of the MR‐PRESSO outlier test to identify outlier SNPs that may cause bias, followed by a re‐evaluation of heterogeneity after removing these outliers and assessing the causal effect. In addition, MR‐PRESSO was employed to detect and correct potential bidirectional outliers. If no outliers were identified, the results from MR‐PRESSO aligned with those from the original IVW analysis. The leave‐one‐out analysis assessed the aggregate effect of the remaining SNPs using the IVW method; if this aggregate effect was consistent with the primary analysis results, it indicated no single SNP had a disproportionate impact on the MR analysis. MR‐Egger intercept was utilized to assess horizontal pleiotropy, with a *p* value less than 0.05 suggesting the presence of pleiotropy and potential bias in IVW estimates. Detailed information on the sensitivity analysis can be found in Table S11.

### 2.5 Statistical Analysis

In the MR analysis, after harmonizing SNPs with the same alleles, we employed the inverse variance weighted (IVW), MR‐Egger, and weighted median (WM) methods for two‐sample MR analysis. The random‐effects IVW method served as the primary statistical model, calculating exposure effects associated with each SNP using the Wald ratio method, followed by a weighted linear regression with a forced zero intercept. Only when more than three SNPs were available were the MR‐Egger and WM methods used as secondary statistical models. MR‐Egger tolerates potential pleiotropy and provides a conservative estimate of the causal effect. The MR‐Egger intercept is a measure of whether there is evidence of pleiotropic effects. If the intercept is significantly different from zero, it suggests the presence of horizontal pleiotropy, violating the assumptions of MR and rendering the analysis results unreliable. Conversely, if the intercept is close to zero, it indicates that the MR assumptions are met, and the results are reliable. The WM method allows reliable causal estimation when less than 50% of the data comes from invalid IVs. Furthermore, when the direction of MR analysis results (OR or *β* values) remained consistent across all three methods (IVW, MR‐Egger, and WM) and the IVW‐calculated *p* value was less than 0.05, the statistical results were considered significant (Wang et al. 2023). When only two SNPs were available, the IVW method was used for analysis, and the Wald ratio method was employed to infer the impact of a single IV on outcomes when only one SNP was available. For presenting the statistical analysis results, OR values and 95% confidence intervals (CIs) were used to present the MR effect estimates for circulating micronutrients on prospective memory. Beta values and their 95% CIs were used to present the MR effect estimates for circulating micronutrients on cognitive performance, fluid intelligence, and reaction time. To address the issue of multiple exposures, a Bonferroni correction significance threshold of 0.003 (0.05/16) was defined. *p* values less than 0.003 were considered statistically significant, while *p* values greater than 0.003 and less than 0.05 were considered to indicate potential association. All analyses were conducted using the “TwoSampleMR” package (version 0.5.8) in R software (version 4.2.2).

## 3 Results

### 3.1 Causal Relationship Between Micronutrients and Reaction Time

**FIGURE 1:**
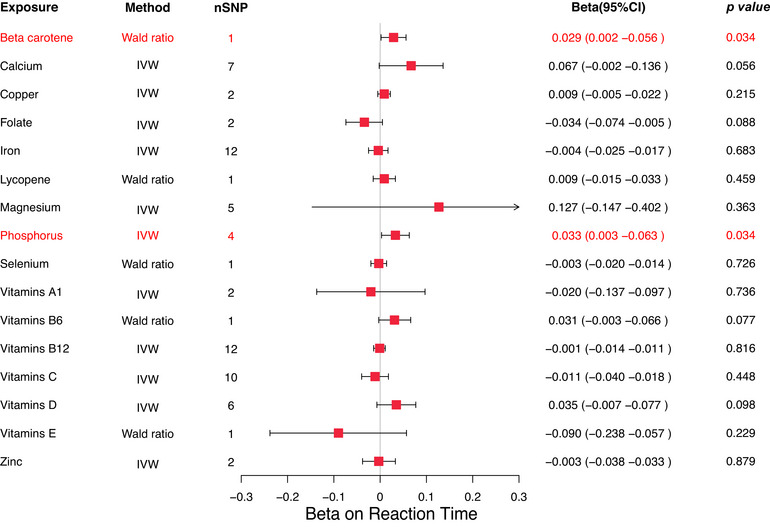
Forest map displaying results of MR studies used in evaluating the potential causal relationship between 16 micronutrients and reaction time.

**FIGURE 2:**
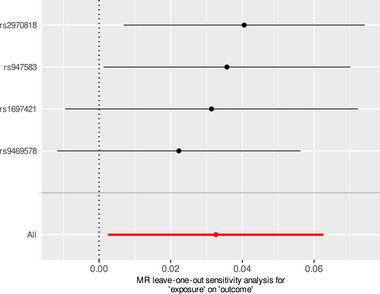
Plots of “leave‐one‐out” method for MR analysis of the causal effect of phosphorus on reaction time showing the sensitivity analysis of the correlation between phosphorus and reaction time.

In the MR analysis of micronutrients and reaction time, our findings suggest that β‐carotene and phosphorus may have a potential positive effect on reaction time, as indicated by shorter reaction times reflecting better cognitive response. Specifically, the Wald ratio estimate for β‐carotene was *β* = 0.029 (95% CI: 0.002–0.056, *p* = 0.034), while phosphorus showed an IVW estimate of *β* = 0.033 (95% CI: 0.003–0.063, *p* = 0.034) (Figure 1). Furthermore, Cochrane's *Q* test revealed no evidence of heterogeneity (*p* = 0.517), and leave‐one‐out analysis confirmed minimal influence of individual SNPs on the causal estimates (Figure 2). MR‐Egger intercept analysis also showed no evidence of horizontal pleiotropy (*p* = 0.533), further demonstrating the robustness of the observed causal relationships. Collectively, these results suggest that higher levels of β‐carotene and phosphorus may be associated with faster reaction times (better cognitive performance).

For other micronutrients, no significant associations with reaction time were observed, suggesting their effects on reaction time might be minimal or unclear (Table S7).

### 3.2 Causal Relationship Between Circulating Micronutrients and Cognitive Performance

**FIGURE 3:**
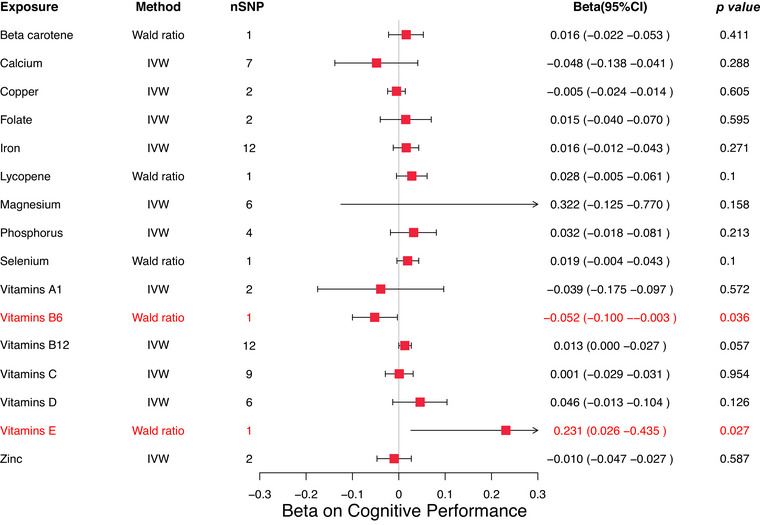
Forest map displaying results of MR studies used in evaluating the potential causal relationship between 16 micronutrients and cognitive performance.

In the MR analysis of micronutrients and cognitive performance, vitamin B6 levels were found to have a potential negative effect, whereas vitamin E levels demonstrated a potential positive effect. Specifically, the Wald ratio estimate for vitamin B6 was *β* = −0.052 (95% CI: −0.100 to −0.003, *p* = 0.036), suggesting that higher levels of vitamin B6 may be associated with poorer cognitive performance. Conversely, the Wald ratio estimate for vitamin E was *β* = 0.231 (95% CI: 0.026–0.435, *p* = 0.027), indicating that sufficient vitamin E intake might improve cognitive function (Figure 3).

For other micronutrients involving multiple SNPs, no significant associations with cognitive performance were detected, suggesting their effects might be minimal or not yet clearly established (Table S8).

### 3.3 Causal Relationship Between Micronutrients and Prospective Memory

**FIGURE 4:**
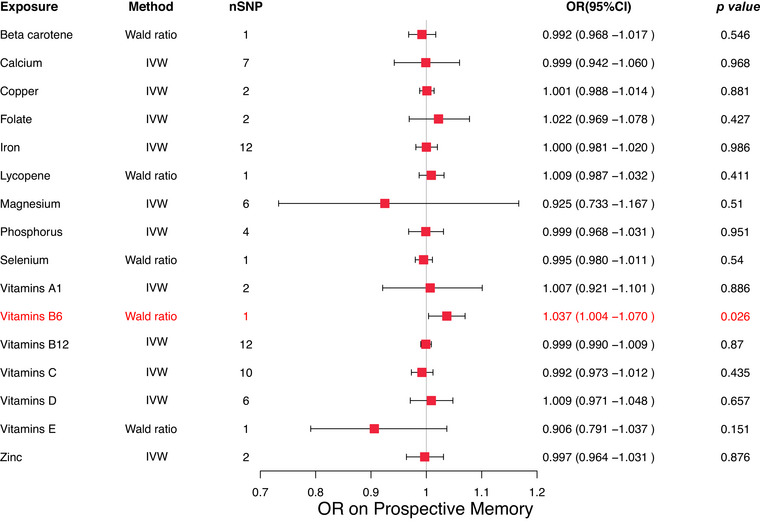
Forest map displaying results of MR studies used in evaluating the potential causal relationship between 16 micronutrients and prospective memory.

In the MR analysis of micronutrients and prospective memory, vitamin B6 was found to have a potential positive association with prospective memory scores (Wald ratio: OR = 1.037, 95% CI: 1.004–1.070, *p* = 0.026). Since higher prospective memory scores indicate poorer performance, this result suggests that elevated levels of vitamin B6 might negatively affect prospective memory (Figure 4).

For other micronutrients, no significant associations with prospective memory performance were observed, suggesting their impact on prospective memory might be minimal or unclear (Table S9).

### 3.4 Causal Relationship Between Circulating Micronutrients and Fluid Intelligence

**FIGURE 5:**
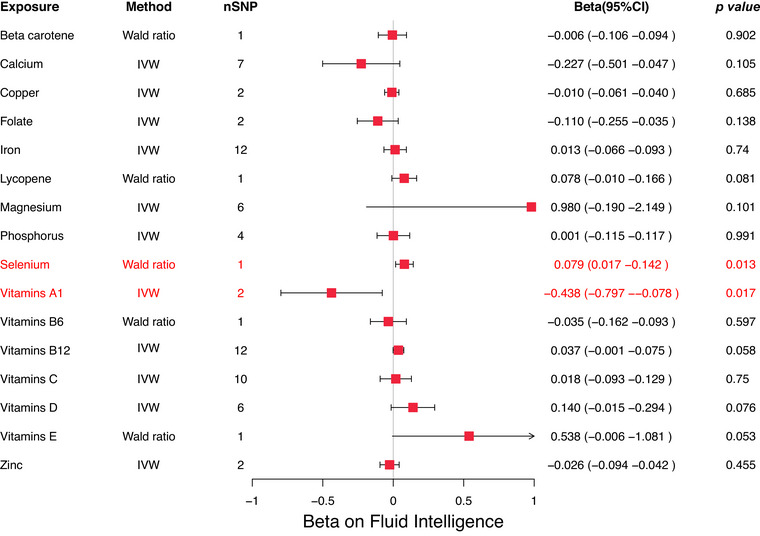
Forest map displaying results of MR studies used in evaluating the potential causal relationship between 16 micronutrients and fluid Intelligence.

In the MR analysis of micronutrients and fluid intelligence, higher selenium concentrations were associated with a potential positive effect, whereas higher vitamin A1 concentrations were associated with a potential negative effect. Specifically, the Wald ratio estimate for selenium was *β* = 0.079 (95% CI: 0.017–0.142, *p* = 0.013), indicating a potential beneficial role in fluid intelligence. Conversely, the Wald ratio estimate for vitamin A1 was *β* = −0.438 (95% CI: −0.797 to −0.078, *p* = 0.017), suggesting that elevated levels of vitamin A1 might negatively affect fluid intelligence (Figure 5).

Cochrane's *Q* test showed no evidence of heterogeneity (*p* = 0.595), suggesting robustness in the findings. For other micronutrients, no significant associations with fluid intelligence were observed, implying that their effects may be negligible or uncertain (Table S10).

## 4 Discussion

This study employed MR to explore causal relationships between micronutrients and cognitive functions, uncovering potential causal effects of specific micronutrients on various cognitive performance metrics. These findings provide novel insights into the mechanisms underlying the role of nutrients in cognitive health.

### 4.1 Potential Mechanisms of Micronutrient Effects on Reaction Time

Our results demonstrate that elevated β‐carotene and phosphorus levels are associated with shorter reaction times, reflecting better cognitive responsiveness. This aligns with prior research. As a precursor to vitamin A, β‐carotene has well‐documented antioxidant and neuroprotective properties (Sies et al. 1992). Its ability to mitigate cognitive impairment through reducing pro‐inflammatory cytokines and improving antioxidant status has been validated in several studies (Zhou et al. 2018; Twitto‐Greenberg et al. 2024). In addition, our findings support earlier reports linking β‐carotene deficiency with increased risks of cognitive impairment and Alzheimer's disease (Twitto‐Greenberg et al. 2024), highlighting its potential as a neuroprotective agent.

Similarly, phosphorus also demonstrated a positive effect in our study, with higher levels associated with faster reaction times. As a critical component of nucleic acids and organic phosphates, phosphorus is essential for maintaining brain function (1990; Subramanian and Khardori 2000). Our findings corroborate previous studies associating low serum phosphorus with higher dementia risk and further suggest that phosphorus may exert its effects by alleviating amyloid‐beta‐related neurotoxicity (Palop and Mucke 2010; Park et al. 2017).

### 4.2 Potential Mechanisms of Micronutrient Effects on Cognitive Performance

Our findings reveal a negative effect of circulating vitamin B6 on cognitive performance, while vitamin E exhibited a positive effect.

Vitamin E, a key antioxidant, has been widely shown to protect the brain from free radical damage. Studies have linked vitamin E deficiency with lipid peroxidation and cognitive impairment (Fukui et al. 2015), and longitudinal research indicates that higher vitamin E intake slows cognitive decline in older adults (Morris et al. 2002). Sufficient vitamin E intake has also been associated with a reduced risk of Alzheimer's disease (Engelhart et al. 2002).

In contrast, elevated levels of vitamin B6 might negatively affect cognitive performance by increasing homocysteine levels, which can induce oxidative stress and neurotoxicity (Selhub 2002; Boot et al. 2003). However, the role of B vitamins remains controversial. Some studies suggest no significant relationship between B‐vitamin intake and Alzheimer's disease risk (Corrada et al. 2005; Morris et al. 2006; Nelson et al. 2009), while others report limited cognitive benefits despite homocysteine reduction and even an increased risk of depression with B‐vitamin supplementation (Aisen et al. 2008). In our study, vitamin B6 exhibited a complex relationship with cognitive performance, warranting further investigation into its underlying mechanisms.

### 4.3 Potential Mechanisms of Micronutrient Effects on Prospective Memory and Fluid Intelligence

The negative effect of vitamin B6 on prospective memory suggests that high levels of vitamin B6 may interfere with memory function, possibly related to its induced neurotoxicity. Previous studies have found that long‐term intake of low‐dose (25–50 mg/d) pyridoxine can lead to neuropathies such as sensory disorders and ataxia (Gdynia et al. 2008; Malet et al. 2020). Excessive use of vitamin B6 may be due to oxidative stress generated by oxygen free radicals, the direct effect of inactive metabolites, and the occurrence of deficiencies in other water‐soluble vitamins (Ghavanini and Kimpinski 2014; Reddy 2022).

For fluid intelligence, increased selenium levels were positively associated with performance, consistent with selenium's antioxidant and neuroprotective roles. Selenium, as a critical component of antioxidant enzymes, reduces oxidative stress and slows cognitive decline (Chen and Berry 2003; Akbaraly et al. 2007; Pitts et al. 2014). Notably, lower selenium levels have been observed in children with learning disabilities (Liu et al. 2022) and are associated with an increased risk of cognitive impairment, particularly in older adults (Shahar et al. 2010).

Conversely, elevated vitamin A1 levels were negatively associated with fluid intelligence, potentially due to the neuroregulatory effects of its metabolite, retinoic acid. While retinoic acid is vital for hippocampal synaptic plasticity at physiological levels, excessive amounts may lead to central nervous system damage (Goldberg 2011; Wołoszynowska‐Fraser et al. 2020). Research also suggests that elevated fetal retinoic acid levels can have teratogenic effects, contributing to neurodevelopmental disorders, especially those involving the cerebellum (Goldberg 2011). Our findings support these mechanisms.

For other micronutrients, no significant associations with cognitive impairment were observed.

### 4.4 Comparisons and Summary

While some micronutrients exhibited potential cognitive effects, the effects of others on reaction time, cognitive performance, prospective memory, and fluid intelligence remain unclear. Possible reasons include:

- The study population predominantly comprised individuals of European ancestry, and sensitivity to micronutrients may vary across populations.
- Observational studies may have failed to control for confounders, yielding false‐positive results and overstating the causal relationships.
- A limited number of available SNPs might have introduced statistical bias.

Overall, our findings provide new evidence for the potential roles of specific micronutrients in cognitive health and underscore the need for further research on micronutrient dosage, metabolism, and long‐term cognitive effects.

### 4.5 Research Significance and Limitations

This study leverages MR to minimize confounding, enhancing the reliability of causal inferences. However, limitations remain. First, we relied on publicly available genomic data, which may not fully account for environmental influences. Second, the mechanisms and dose–response relationships of specific micronutrients warrant further investigation. Third, potential gender differences in the associations between micronutrients and the outcomes we studied were not explored. This lack of analysis regarding gender differences represents a limitation and could be an important aspect for future research in this area.

## 5 Conclusion and Implications for Clinical and Public Health

**FIGURE 6:**
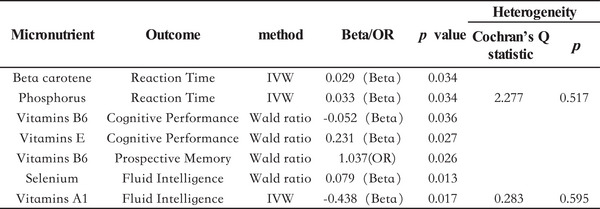
The summary of the statistically significant findings obtained in this study.

This study supports the potential benefits of appropriate supplementation with antioxidants (e.g., β‐carotene, phosphorus, vitamin E, and selenium) for cognitive health while cautioning against excessive intake of certain micronutrients (e.g., vitamin A1 and vitamin B6). These findings provide a scientific basis for nutrition‐based cognitive health interventions. The summary of the statistically significant findings obtained in this study is shown in Figure 6.

Previous studies have shown that cerebrovascular dysfunction is a key factor leading to cognitive decline. Endothelial colony‐forming cells (ECFCs) (Negri et al. 2025) and time‐restricted eating (TRE) (Milan et al. 2024) are of great value in the treatment of cerebrovascular dysfunction. ECFCs can promote angiogenesis and improve the functional state of cerebral blood vessels. TRE, on the other hand, can have a positive impact on cerebral blood vessels by regulating the body's metabolic rhythm, thus preventing cognitive decline to a certain extent.

In future research, we should conduct multi‐dimensional combined studies on the findings of micronutrients in this study, ECFCs, TRE, and other factors. We need to comprehensively consider their synergistic mechanisms on cognitive health and explore how to more effectively prevent and improve cognitive impairment through combined intervention measures such as optimizing nutritional supplementation, regulating the function of ECFCs, and rationally arranging TRE. This is expected to provide more comprehensive and targeted intervention strategies and methods in the field of cognitive health and offer stronger support for safeguarding people's cognitive health.

## Author Contributions

**Sen‐fu Yuan**: conceptualization, investigation, visualization. **Chang‐hai Long**: conceptualization, software, methodology. **Xu Zhang**: data curation, formal analysis. **Mi Yuan**: project administration, writing–original draft. **Jun Li**: writing–original draft, validation. **He‐gang Wu**: writing–original draft, writing–review and editing, resources, funding acquisition, supervision.

## Ethics Statement

Since we adopted publicly available data for this study, ethical approval and informed consent were not required.

## Conflicts of Interest

The authors declare no conflicts of interest.

### Peer Review

The peer review history for this article is available at https://publons.com/publon/10.1002/brb3.70488

## Supporting information



Supporting Information.

## Data Availability

The data that supports the findings of this study are available in the supplementary material of this article.
